# “Masato de Yuca” and “Chicha de Siete Semillas” Two Traditional Vegetable Fermented Beverages from Peru as Source for the Isolation of Potential Probiotic Bacteria

**DOI:** 10.1007/s12602-021-09836-x

**Published:** 2021-08-27

**Authors:** Teresa D. Rebaza-Cardenas, Kenneth Silva-Cajaleón, Carlos Sabater, Susana Delgado, Nilda D. Montes-Villanueva, Patricia Ruas-Madiedo

**Affiliations:** 1 Department of Microbiology and Biochemistry of Dairy Products, Instituto de Productos Lácteos de Asturias – Consejo Superior de Investigaciones Científicas (IPLA-CSIC), Villaviciosa, Asturias Spain; 2 Facultad de Ingeniería Agraria, Universidad Católica Sedes Sapientiae (UCSS), Lima, Peru; 3 Group Functionality and Ecology of Beneficial Microbes, Instituto de Investigación Sanitaria del Principado de Asturias (ISPA), Asturias Oviedo, Spain

**Keywords:** Vegetable, Fermented beverage, Food, Probiotic, Lactic acid bacteria, Typing, HT29

## Abstract

In this work, two Peruvian beverages “Masato de Yuca,” typical of the Amazonian communities made from cassava (*Manihot esculenta*), and “Chicha de Siete Semillas,” made from different cereal, pseudo-cereal, and legume flours, were explored for the isolation of lactic acid bacteria after obtaining the permission of local authorities following Nagoya protocol. From an initial number of 33 isolates, 16 strains with different RAPD- and REP-PCR genetic profiles were obtained. In Chicha, all strains were *Lactiplantibacillus plantarum* (formerly *Lactobacillus plantarum*), whereas in Masato, in addition to this species, *Limosilactobacillus fermentum* (formerly *Lactobacillus fermentum*), *Pediococcus acidilactici*, and *Weissella confusa* were also identified. Correlation analysis carried out with their carbohydrate fermentation patterns and enzymatic profiles allowed a clustering of the lactobacilli separated from the other genera. Finally, the 16 strains were submitted to a static in vitro digestion (INFOGEST model) that simulated the gastrointestinal transit. Besides, their ability to adhere to the human epithelial intestinal cell line HT29 was also determined. Following both procedures, the best probiotic candidate was *Lac. plantarum* Ch13, a robust strain able to better face the challenging conditions of the gastrointestinal tract and showing higher adhesion ability to the intestinal epithelium in comparison with the commercial probiotic strain 299v. In order to characterize its benefit for human health, this Ch13 strain will be deeply studied in further works.

**Supplementary Information:**

The online version contains supplementary material available at 10.1007/s12602-021-09836-x.

## Introduction

Beverages obtained from fermented vegetables have a long tradition of consumption in many ancestral cultures, such as those of Latin America, where nowadays some of them are still widely handcraft-manufactured being part of the sustainable economy of most families living in specific regions [1, 2]. These fermented foods and beverages have an undoubted high nutritional value; also, the microbial transformation of the raw material favors the occurrence of health-promoting components and metabolites [3]. In fact, it has been proposed that the controlled fermentations of ancient protein-rich plants could be a tool to search for alternatives of protein sources [4]. However, we should keep in mind that these approaches must be performed in a sustainable way, ensuring the access of these traditional food sources to the local population who have kept the ancestral seeds and their uses. This has been enclosed within the objectives of the “Convention on Biological Diversity” dealing to conserve the biological diversity and its sustainable use, as well as to ensure the equitable sharing of the benefits that can be obtained for the use of the genetic resources; the last objective has been implemented through the Nagoya Protocol [5].

These artisanal foods are a natural source for the isolation of novel microorganisms, since they are obtained through spontaneous, non-controlled fermentations, thus keeping the biodiversity of the natural microbiota; then, they constitute a valuable source of microbial diversity, ready to be explored [6, 7]. However, in spite of the high potential of these heritage-fermented foods, studies about their microbiota and their genetic diversity are scarce or non-existent in some cases. One of the microbial groups that are mostly always present in fermented products are the lactic acid bacteria (LAB), which are the main drivers of lactic fermentation. In recent years, special attention has been payed to the isolation and characterization of LAB from different plant-based foods in the Latin American geography, such as “Tocosh” and “Tunta” from potato in Peru [8, 9], Chicha from maize in Argentina [10], or fermented “Chia” also in Argentina [11], to cite some of them. A wide variety of LAB genera have been described, although members *Lactobacillus* spp. are often found; this genus has been recently reclassified and some species renamed [12]. The reason behind the interest in the LAB is the multiple applications of this bacterial group, not only as a way to standardize and improve the hygienic-sanitary quality of the fermented products, but also as drivers of the “identity signature” of foods with geographical indications (GIs). In this regard, it has been proposed that the microbial resource information could be integrated into the context of the fermented food GIs [13]. LAB are able to modify components of the food matrix, which might improve their bioaccessibility and digestibility, thus increasing the nutritional values [14]; but, also the metabolites resulting from the bacterial activity are responsible for the aroma, flavor, viscosity, and texture of the fermented product [15]. Besides, some specific strains are able to produce other molecules along the fermentations, such as vitamins, neuroactives, bioactive peptides, bacteriocins, among others, that might have an influence on our health [16]. In this regard, some of the LAB having positive effects on our well-being are known as probiotics, which have been defined by FAO-WHO in 2001 as “live microorganisms that, when administered in adequate amounts, confer a health benefit on the host” [17]. The probiotics intended for food (and feed) applications must be safe for human (or animal) consumption; therefore, only certain species are Generally Recognized as Safe (GRAS, according to FDA administration), or are included in the Qualified Presumption of Safety (QPS) list [18]. Among them, the most common belong to genera *Bifidobacterium* and *Lactobacillus* and there are multiple mechanisms by which probiotic strains might exert the beneficial action. The positive effect on health must have strong scientific evidence behind, before being used for clinical applications [19] or being included in the formulation of foods with health claims. In relation to this, it has been recently reported the differences among the concept probiotic, fermented food, and probiotic fermented food. Terms including “probiotic” need to have evidence of health benefits; besides, in the “probiotic fermented food,” the fermentation can be conducted by the probiotics, or the fermented food could contain probiotics, but is always a food format [20].

The objective of the present study was to isolate new LAB from two typical fermented beverages of Peru, “Masato de Yuca” (from *Manihot esculenta*) and “Chicha de Siete Semillas,” a poli-cereal mixture combined with legumes and aromatic plants. Furthermore, the selection of the most robust strains, able to survive to the gastrointestinal transit and to transitionally adhere to the colon, will be achieved. Following this “roadmap,” the best candidates will be selected to undertake future studies about their potential probiotic capabilities. This work was conceived in the framework of the ProInfant-CYTED project, a multidisciplinary and collaborative work of several Latin American, Spanish, and Italian research groups, in which the final long-term aim is to develop high nutritional value, vegetable-based fermented foods, containing well-adapted probiotic strains able to reduce malnutrition-related diseases, such as diarrhea and respiratory infections.

## Material and Methods

### Food Sample Collection

Two types of traditional Peruvian fermented foods were used to search for LAB (Table 1). The “Chicha de Siete Semillas” (hereafter referred to as Chicha) was collected from 5 producers located in the province of Huanta (Ayacucho Department) which sell their products on specialized cellars (“bodegas”), whereas the “Masato de Yuca” (hereafter referred to as Masato) was obtained from 6 producers that offer their products in local markets or street selling in the provinces of Chanchamayo and Satipo, both belonging to the Peruvian Amazonia. The collection of the food samples was performed following the Nagoya protocol, with the corresponding permission of the local Peruvian authorities (Contract No. 001 2018-MINAGRI-SERFOR/DGGSPFFS-DGSPF). For LAB isolation, serial dilutions were made in 0.1% (w/v) of peptone water (HiMedia, India) which were spread in the surface of agar-MRS (pH 6.4, Conda, Spain) supplemented with 50 μg/mL cycloheximide to prevent fungi overgrowth. Plates were incubated at 32 °C for 48–72 h. Afterwards, colonies of different morphology were picked up to perform a Gram staining; those Gram-positives were screened for the absence of catalase activity and spore formation, using standard procedures. The final isolates were stored at − 20 °C in MRS broth containing 30% glycerol. Samples were sent to IPLA-CSIC directly inoculated from stocks by puncture into tubes containing MRS with 0.75% agar.

**Table 1:** Geolocation (Universal Transverse Mercator coordinates) and characteristics of the fermented foods “Masato de Yuca” and “Chicha de Siete Semillas” analyzed in this study

Artisanal producer: “Masato de Yucca”	Geolocation: Province, District (UTM)			Ingredients (flour)			pH food
				Tuber			
M0	Satipo, 18L (0,539,952; 8,755,970)			Yucca (*Manihot esculenta*)			4.15
M2	Chanchamayo, Pichinaqui, 18L (0,515,984; 8,790,091)			Yucca			4.05
M3	Chanchamayo, Pichinaqui, 18L (0,516,242; 8,789,960)			Yucca			4.06
M4	Chanchamayo, Pichinaqui, 18L (0,516,452; 8,789,818)			Yucca			3.95
M7	Chanchamayo, Perené, 18L (0,510,651; 8,794,800)			Yucca			3.99
M8	Chanchamayo, Pichinaqui, 18L (0,513,931; 8,792,256)			Yucca			4.27
Artisanal producer: “Chicha de Siete Semillas”	Geolocation: Province, (UTM)	Ingredients (flours, condiments)					pH food
		Cereal	Pseudo-cereal		Legume	Condiments	
CH1- “La Verídica”^a^	Huanta, 18L (331,130, 8,693,818)	Maize (yellow), maize “jora”^b^, barley	“Quinua”^c^, “kiwicha”^d^		Chickpea, “habas”^e^	Chamomile, “cedrón”^f^, apple peal	3.57
CH2- “La Auténtica”	Huanta, 18L (582,038, 8,568,829)	Maize “morocho”^g^, barley, wheat	“Quinua”		Chickpea, “habas,” vetch	Chamomile, “cedrón,” anise, peal (apple, banana, orange), “chancaca”^h^	3.39
CH3- “Real Calidad”	Huanta, 18L (582,001, 8,568,821)	Maize “morocho,” barley, wheat	“Quinua”		Chickpea, “habas,” vetch	Chamomile, “cedrón,” “chancaca	3.44
CH4- “La Palmera”	Huanta, 18L (582,008, 8,568,815)	Maize “morocho,” “jora” (black), barley, wheat	“Quinua,” “kiwicha”		Chickpea, “habas”	Chamomile, “cedrón,” anise	3.79
CH5- “Chicha”	Huanta, 18L (582,011, 8,568,808)	Maize (white), barley, wheat	“Quinua,” “kiwicha”		Chickpea, “habas”	-	3.71

^a^Name of the cellar that sell the “Chicha”

^b^“jora” is a malted corn

^c^“quinua” or quinoa

^d^“kiwicha” *Amaranthus caudatus*

^e^“habas” or kidney beans

^f^“cedrón” or “hierva luisa” is lemon verbena (*Aloysia citrodora*)

^g^“morocho” is a hard-yellow maize

^h^“chancaca” is whole cane sugar

### Isolation and Identification of LAB

A collection of 33 potential LAB isolates (20 from Chicha and 13 from Masato) were obtained; at their arrival at IPLA-CSIC, they were spread on the surface of agar-MRS plates (Biokar Diagnostics, France) to check purity and to prepare new stocks which were stored at − 80 °C with the same cryoprotectant. This bacterial collection was identified by sequencing the 16S rRNA gene using the primers 27F (5′-AGAGTTTGATCCTGGCTCAG-3′) and 1492R (5′-CTACGGCTACCTTGTTACGA-3′) [21]. Previously, DNA was purified from overnight cultures in MRS broth using the GenElute Bacterial Genomic DNA kit (Sigma-Aldrich, USA) following the manufacturer instructions with modifications; this, consisted on an enzymatic treatment with lysozyme (45 mg/mL) (Merck, Germany) and mutanolysin (5U, Sigma-Aldrich) as previously reported [22]. The PCR reaction mixture contained the following (in a final volume of 25 μL): 12.5 μL of Taq DNA Polymerase Master Mix (Ampliqon, Denmark), 0.6 μL of each primer (10 μM), 1 μL of DNA as template, and 10.3 μL of molecular grade water (Sigma-Aldrich). The PCR conditions were as follows: one cycle of 95 °C for 5 min, 35 cycles of 95 °C for 30 s, 55 °C for 30 s, and 72 °C for 5 min, ending with a final extension step of 72 °C for 7 min. PCR reactions were performed in the UnoCycler thermocycler (VWR-Avantor, Spain). Amplified PCR products were sequenced at Macrogen Spain Inc. (Spain) and sequences were compared with those held in the NCBI database using the BLASTn tool; the species level was assigned given that the percentage of nucleotide identity obtained was equal or higher than 99.5%.

### LAB Typing

Two bacterial PCR-typing techniques were applied in order to determine the genomic fingerprints of the isolates. RAPD (randomly amplified polymorphic DNA) was carried out using the primer M13 (5′-GAGGGTGGCGGTTCT-3′) [23] in a final reaction mixture of 50 μL containing 25 μL of Taq DNA Polymerase Master Mix, 10 μL of primer M13 (10 μM), 5 μL of purified DNA, and 10 μL of molecular grade water. PCR conditions were the following: an initial step of 95 °C for 5 min, 40 cycles of 94 °C for 60 s, 42 °C for 20 s, and 72 °C for 2 min, and a final extension step of 72 °C for 10 min. REP (repetitive extragenic palindromic sequences) was performed with the primer BOX-A2R (5′-ACGTGGTTTGAAGAGATTTTCG-3′) [24]. The PCR mixture (in 50 μL of final volume) was as follows: 25 μL of Taq DNA Polymerase Master Mix, 5 μL BOX-A2R primer (10 μM), 2 μL of DNA, and 18 μL of molecular grade water, which was subjected to the next PCR conditions: an initial step of 95 °C for 7.5 min, 30 cycles of 90 °C for 30 s, 40 °C for 60 s, and 72 °C for 2 min, and a final extension 72 °C for 10 min. All primers used in this work were synthesized at Macrogen. RAPD-PCR and REP-PCR products (10 μL) were mixed with 1 μL of EZ-vision DNA Dye (VWR-Avantor) and loaded into agarose gels (1.5% w/v, in TAE buffer) to carry out the electrophoresis (at 75 V for 80 min). Afterwards, gels were visualized and photographed in a GBox (Syngene Europe, UK). The GeneRuler™ DNA Ladder Mix (Thermo Scientific, USA) from 100 to 10,000 bp was used as a DNA size marker.

The carbohydrate fermentation profile of the 33 isolates was determined by means of the Api-CH50 colorimetric assay (bioMérieux, Spain), whereas the presence of specific enzymatic activities was assessed for the 16 selected strains with the Api-Zym gallery (bioMérieux). In both cases, the manufacturer’s instructions were followed.

### Static In Vitro Simulation of Gastrointestinal Transit of Selected LAB

The capability of the 16 strains selected in this study, together with the probiotic *Lac. plantarum* DSM9843 (commercial name 299v), to survive to the gastrointestinal transit was evaluated following the INFOGEST-consensus static in vitro food digestion procedure [25, 26] with some modifications. Standardized cultures of the strains were obtained as follows: stocks (stored at − 80 °C) were spread over the surface of agar-MRS and incubated for at least 2 days at 32 °C; one single colony was inoculated in MRS broth and overnight cultivated to inoculate (2%) fresh medium that was grown for 18 ± 1 h. These standardized cultures were washed ones with NaCl 0.85% and suspended in the same volume of 11% (w/v) reconstituted skim milk (BD-Difco, Thermo Fisher Scientific, Spain) that was previously heat-treated (two times at 90 °C for 10 min, with an overnight step of maintenance at room temperature). The bacterial suspensions in milk were sequentially submitted to the oral, gastric, duodenal, and intestinal challenges. Bacterial counts (CFU/mL) in each step, as well as the initial number of viable in the bacterial suspension, were carried out by preparing serial dilutions in Ringer ¼ (Merck) and plating in agar-MRS plates that were incubated for 48 h at 32 °C. This experimental setup was performed in triplicate for each strain. The percentage of survival was calculated as the CFU/mL obtained in each step with respect to the initial CFU/mL.

### Adhesion to HT29 Epithelial Cells of Selected LAB

The adhesion of the 16 selected strains, and the probiotic strain DSM9843, to the human intestinal epithelial was evaluated using the cell line HT29 (ECACC 91,072,201, European Collection of Cell Cultures, UK). The maintenance conditions and the adhesion procedure were performed as previously described [27]. In short, bacterial suspension from standardized cultures was washed with PBS, diluted 10 times (to obtain about 1 × 10^8^ CFU/mL), and resuspended in McCoy’s medium (Sigma) supplemented with 10% fetal bovine serum (Sigma) and 3 mM L-glutamine (Sigma). Then, HT29 cell monolayers (previously cultivated for 11 days at 37 °C/5% CO_2_ in 24-well plates) were carefully washed twice with Dulbecco PBS (Sigma) before adding 0.5 mL of each bacterial suspension in the complete McCoy’s medium (ratio HT29:bacteria, 1:10). Plates were incubated under the same condition for 1 h; afterwards, supernatants were removed and the monolayers washed twice to remove the unattached bacteria. Finally, 0.25 mL of 0.25% EDTA-trypsine (Sigma) was added to desegregate the HT29 monolayer and incubated for 5–10 min at 37 °C/5% CO_2_; the reaction was stopped by adding equal volume of complete McCoy’s medium. This experimental procedure was repeated, at least, in triplicate per each strain. Bacteria initially added and those recovered after the adhesion procedure were enumerated by plating serial dilutions made on Ringer ¼ in agar-MRS. The percentage of adhesion was calculated as CFU/mL of bacteria adhered, with respect to the CFU/mL of bacteria added.

### Statistical Analysis

The quantitative data were analyzed by means of one-way ANOVA followed by the S–N-K (Student–Newman–Keuls) mean comparison test to determine the differences among the 16 strains characterized in this study; this was performed using the IBM-SPSS statistics for Window version 26.0 (IBM Corp., USA). Qualitative data from enzymatic activities and carbohydrate fermentation profiles was integrated using two different types of computational models: (i) DIABLO biomarker discovery pipeline [28] to determine characteristic fermentation profiles of *Lim. fermentum* and *Lac. plantarum*, considering 2 components and 5 variables selected on each component; and (ii) hierarchical all-against-all association testing (HAIIA) (http://huttenhower.sph.harvard.edu/halla, last accessed: 21/09/2020) of enzyme activity and carbohydrate fermentation data, considering *q*-values and a Bonferroni False Discovery Rate of 0.05. In addition, principal component analysis (PCA) was performed to illustrate sample distribution according to the producer. These models were computed on R v3.5.0.

## Results and Discussion

### Traditional Uses of Vegetables as Peruvian Intangible Cultural Heritage

The consumption of fermented foods and beverages has been dated from ancient times worldwide. It has been also documented that it was the traditional way to preserve raw materials for survival, once that humans became to domesticate animals and being farmers in the Neolithic [29, 30]. Regarding Latin America, there are pieces of evidence of local resources uses from pre-Hispanic societies. As an example, maize (*Zea mays*) consumption in the south-central Andes was evidenced analyzing archeological potsherds, ca. 3rd to 16th AD, using δ^13^C and molecular techniques to study the fatty acid compositions of the residues [31]. As occurred in other world civilizations, the collection and storage of the vegetable and animal raw materials in Latin American areas favored the spontaneous fermentations driven by their natural microbiota. Therefore, a wide variety of fermented food and beverages were, and currently are, elaborated following traditional usages [1].

In the Peruvian case, there is scarce information in the scientific literature about the manufacture practices of some of their traditional products [32]. In the case of the two beverages studied in our work, a photographic study about the “Masato de Yuca” manufacture was recently reported [33]; this was coincident with the process flow diagram (see supplementary Fig. S1) collected from the communities of Chanchamayo and Satipo that contributed to our study, although in our case commercial samples were collected which did not use human saliva for their preparation (Table 1). As far as we could find, there is no previous information in scientific databases about the elaboration of “Chicha de Siete Semillas,” a multi-cereal, pseudoceral, and legume fermented beverage (supplementary Fig. S1). However, this process could be, so far, similar to that recently reported for “Chicha de Jora” [7].

Independently of beverage, there are common steps in the production of both products, given that the raw material needs to be processed in order to improve the digestibility of the vegetables. In the context of our study, the most interesting step is the fermentation, which will be carried out by the natural microbiota that survived the previous processing, or by that introduced from the manufacturer’s environment, including that of the human origin. Yeasts and LAB will be the main actors involved in alcoholic and lactic acid fermentations, respectively. Some of the traditional Latin American fermented foods have been screened as a source for the isolation and characterization of LAB due to the biotechnological potential of this microbial group [34, 35].

### Traditional Fermented Vegetables as Source of LAB

The fermented beverages “Masato de Yuca” and “Chicha de Siete Semillas” manufactured in a traditional way by 6 and 5 different producers, respectively, were used to isolate autochthonous LAB. A collection of 33 isolates, 13 from Masato and 20 from Chicha, were directly obtained from the fermented beverages, without any enrichment step, in cycloheximide-agar-MRS (Table 2). The 16S rRNA gene sequencing of the isolates obtained from the Chicha revealed that all belonged to the species *Lactiplantibacillus plantarum* with a degree of homology, with sequences held in the NCBI database, higher than 99%. This species was also isolated in the highest abundance in Masato; but, two isolates of *Limosilactobacillus fermentum*, one of *Pediococcus acidilactici*, and one *Weissella confusa* were also identified. As far as we could have found, there are no previous data in the scientific literature about the LAB diversity of Chicha made with, at least, seven ingredients. However, a previous study of the LAB diversity of “Masato de Yuca” made from cassava, including those elaborated with human saliva, showed the predominance of *Lac. plantarum*, followed by *Lactobacillus alimentarius* (currently *Companilactobacillus alimentarius*) and *Lactobacillus acidophilus* [36]. Recently, it has been published a wide repertoire of fermented beverages elaborated from this tuber by indigenous communities from South America such as “chicha,” “calugi,” “yakupa,” “caxiri,” “cauim,” “tarubá,” or “parakari,” and in all of them different lactobacilli were isolated most from *Lac. plantarum* species [37]. In fact, this species was found also among the most abundant bacteria by means of a culture-independent technique (PCR-DGGE) in a single “tarubá” product elaborated by a tribe in the Brazilian Amazonia [38]. In this point, we must remark that the reduced number of samples analyzed in the study of our Peruvian beverages does not allow having an overall picture of the biodiversity of the microbiota present in these traditional fermented foods. *Lac. plantarum* is often isolated from vegetable-based fermented foods, as well as from other animal sources such as dairy products [22]. Indeed, *Lac. plantarum* is the species most often found in plants and plant-derived foods, and it can be considered as “generalist” species able to occupy a wide range of ecological niches [39]. This could be related to its genomic versatility and plasticity given that it is able to acquire or lose different genetic features linked to the environmental requirements, thus being considered a “natural metabolic engineer” [40]. Besides, *Lac. plantarum* is highly specialized in carbohydrate utilization, as it was observed after the comparative genome analysis of several strains [41]. However, curiously, *Lac. plantarum* was not predominant species in potato-based fermented “Tunta” [9] and “Tocosh” [8] both elaborated from communities of the “Altiplano Andino” (high Andean regions more than 2300 m above sea level) in Peru. *Lim. fermentum*, together with *Lac. plantarum*, was also abundantly isolated from maize-based fermented foods from Kenya [42]. Both lactobacilli, together with representatives of other LAB, were frequently isolated from traditional fermented foods from Latin America, such as maize-based “Chicha” [10] and “Chia” sourdough [11], both from Argentina. Traditional fermented foods from other geographical worldwide areas also reported the isolation of lactobacilli species in high abundance, among the LAB; to cite some examples, those isolated from water after cassava fermentation in Cameroon (Central Africa) [43] or from “Laphet,” a traditional tea leaf (*Camellia sinensis*) fermented food in Myanmar (Southeast Asia) [44].

**Table 2:** Identification of the lactic acid bacteria isolates from the traditional Peruvian fermented beverages “Chicha de Siete Semillas” (20 isolates) and “Masato de Yuca” (13 isolates) after comparing their 16S rDNA sequences with those in Genbank NCIB database. Isolates with similar DNA band pattern (see supplementary Fig. S2) after typing are indicated

Code	Identification	% identity	Genbank accession no	Isolates with similar DNA pattern profile
**“Chicha de Siete Semillas”**				
Ch13	*Lac. plantarum*^a^	99.85%	MW322969	Ch13
Ch17	*Lac. plantarum*	99.85%	MW322970	Ch17, Ch31
Ch23	*Lac. plantarum*	99.71%	MW322971	Ch23
Ch33	*Lac. plantarum*	100%	MW322972	Ch33, Ch34, Ch11, Ch12, Ch14, Ch16, Ch21, Ch22
Ch41	*Lac. plantarum*	99.85%	MW322973	Ch41,
Ch43	*Lac. plantarum*	99.85%	MW322974	Ch43, Ch42, Ch52, Ch32
Ch51	*Lac. plantarum*	100%	MW322975	Ch51, Ch15, Ch18
**“Masatto de Yuca”**				
M01	*Lac. plantarum*	100%	MW322976	M01
M04	*Lac. plantarum*	100%	MW322977	M04
M21	*Lac. plantarum*	100%	MW322978	M21, M22
M41	*Lac. plantarum*	100%	MW322979	M41
M82	*Lac. plantarum*	100%	MW322980	M82
M02	*Lim. Fermentum*^b^	99.85%	MW322981	M02
M31	*Lim. fermentum*	99.71%	MW322982	M31
M71	*Pediococcus acidilactici*	99.56%	MW322983	M71, M03, M32, M33
M81	*Weissella confusa*	100%	MW322984	M81

^a^*Lactiplantibacillus plantarum*

^b^*Limosilactobacillus fermentum* (according to [12])

In order to determine the genetic variability among the 33 isolates, the RADP- and REP-PCR fingerprints with M13 and BOX-A2R primers, respectively, were obtained (see supplementary Fig. S2, Table 2). The 20 *Lac. plantarum* isolates obtained from Chicha were assigned to seven, clearly distinguishable, different RAPD-PCR profiles, while the REP-PCR technique was less discriminative since no clear differences were detected for the couples Ch13, Ch17 and Ch41, Ch43, each from the same producer (1 and 4, respectively). The 6 *Lac. plantarum* isolated from Masato presented five different profiles and, in this case, the primer BOX-A2R allowed the clear distinction between strains M41 and M81. In this regard, we have previously observed that the primer M13 was better than BOX-A2R to reveal different fingerprints among exopolysaccharide-producing *Lac. plantarum* isolated from Algerian traditional dairy products [45]. The 2 *Lim. fermentum* also showed different band patterns with both primers. However, it seems that none of these techniques help to distinguish among the four *P. acidilactici* isolated from Masato of three different producers (0, 3, and 7). Finally, we have observed higher genetic variability among the *Lac. plantarum* isolated from Masato than from Chicha, in spite of the most complex formulation of the last beverage with respect to the former (Table 1). This variability could be expected given that the two beverages were obtained after spontaneous fermentations of the plant substrates, without black-sloping inoculation; then, the degree of LAB “domestication” is lower [46].

In addition to the genetic typing, the carbohydrate fermentation pattern assessed by means of ApiCH50 (Table S1) and the enzymatic activities measured by Api-zym (Table S2) were determined for the isolates under study. Biological associations between both parameters, were performed by means of the HAllA test (Supplementary material Fig. S3). As a result, statistically significant (*p*_adj_ < 0.05) positive (red cells) and negative (blue cells) associations were determined revealing a high degree of correlation between the enzyme profiles of each strain and their carbohydrate metabolism. In general, bacteria showing acid phosphatase and esterase lipase (C8) activities were not able to metabolize D-galactose, D-ribose, D-fructose, and amygdalin. In contrast, valine arylamidase, β-glucosidase, and N-acetyl-β-glucosaminidase activities were found in those bacteria that were able to consume these substrates, indicating a positive relationship between these enzymes and carbohydrate structures. Moreover, the presence of these activities (i.e., valine arylamidase, β-glucosidase, and N-acetyl-β-glucosaminidase) was also associated with the capacity for metabolizing a wide range of carbohydrate substrates including L-arabinose, D-sorbitol, D-manitol, methyl-a-D-mannopyranoside, D-raffinose, and arbutin. On the other hand, a positive correlation was found between leucine arylamidase activity and the fermentation of N-acetyl glucosamine. Similarly, the presence of β-galactosidase activity showed a slightly positive correlation to glycerol and D-arabitol fermentation. Finally, those bacteria showing esterase (C4) activity were not generally able to hydrolyze D-galactose, D-ribose, D-fructose, and amygdalin. On the other hand, it could be considered that strains isolated from traditional Peruvian fermented foods obtained from different producers may show relevant differences in their metabolic capabilities. To better discriminate between strains from several producers, PCA analysis was performed (Supplementary material Fig. S4). In general, strains from producers CH2, CH3, and M2, exerting β-galactosidase, N-acetyl-b-glucosaminidase, β-glucosidase, and valine arylamidase activities, did not show any relevant α-galactosidase and α-glucosidase activity (Supplementary material Fig. S4A). With regard to carbohydrate fermentation (Supplementary material Fig. S4B), two major groups were observed: those strains consuming D-turanose, D-raffinose, arbutin, D-sorbitol, and D-galactose, and those that were not able to metabolize these substrates. In addition, strains from CH1 were characterized by the consumption of D-turanose while strains from the CH2 producer showed the highest affinities for D-raffinose. Strains from the M4 producer were mainly characterized by the consumption of D-galactose. Therefore, it was possible to associate the metabolic capabilities of the LAB isolated from different producers, which could be related to the specific manufacturer’s practices of each one.

**Fig. 1:**
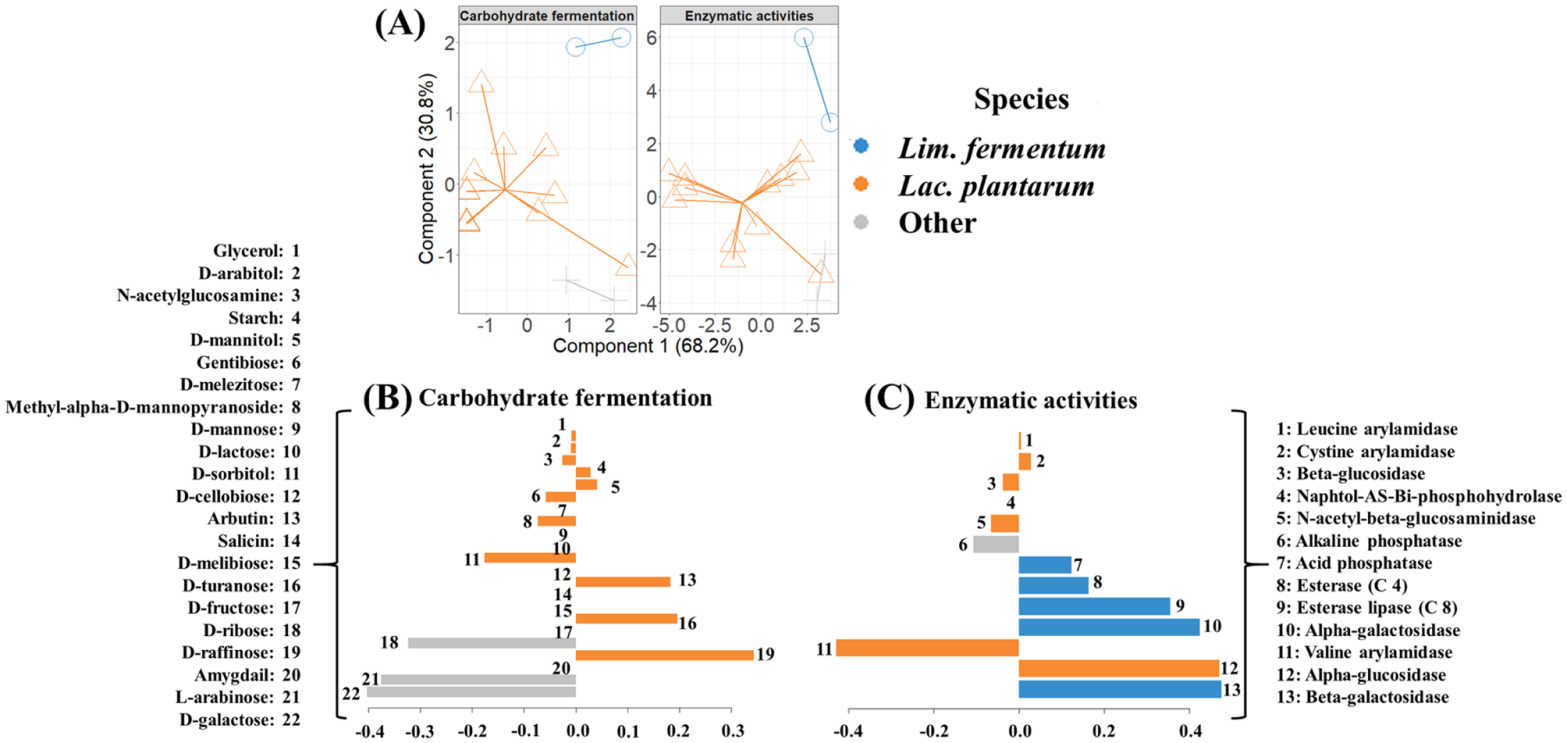
(**A**) Differential patterns in carbohydrate fermentation and enzymatic activities according to the species used (*Lim. fermentum*, *Lac. plantarum*, and other species) obtained by DIABLO pipeline. Characteristic carbohydrates fermented (**B**) and enzymatic activities (**C**) for each species were calculated and expressed as variable importance coefficients, showing their positive or negative influence. Missing bars indicate that no relevant effect was observed for that variable on any species group

Finally, data integration from LAB phenotyping allowed establishing characteristic profiles for *Lim. fermentum* and *Lac. plantarum* strains (Fig. 1A), which could not be inferred using a single dataset (i.e., enzymatic activities or carbohydrate fermentation only). In addition to the high percentage of variance explained by the two components (Fig. 1A), the areas under the receiver operating characteristic (ROC) curves, a common method to express model performance that is insensitive to class imbalance, were calculated to ensure model quality. Areas under ROC curves were above 0.9 in all cases, indicating an excellent discrimination of characteristic profiles of *Lim. fermentum*, *Lac. plantarum*, and other species. In general, strains from *Lac. plantarum* were characterized by a high capacity to metabolize D-raffinose, D-turanose, and arbutin while strains from genera, other than Lim. fermentum and *Lac. plantarum*, led to a higher hydrolysis of D-galactose, L-arabinose, and D-ribose than *Lim. fermentum* (Fig. 1B). With regard to enzymatic activities of both genera, *Lac. plantarum* was characterized by high α-glucosidase while *Lim. fermentum* exerted high α-galactosidase, β-galactosidase, and esterase lipase C8 activities (Fig. 1C). Other types of bacteria included in this study, especially *P. acidilactici*, were mainly characterized by low alkaline phosphatase activity. Therefore, after the phenotypic and genetic analyses, we have selected 16 strains, showing different traits, for further studies. Given that they were isolated from non-controlled fermentations, in geographic areas that conserve high biological diversity, we consider that these wild isolates could have a great biotechnological potential [15].

### Selection of Robust Candidates Well-adapted to Vegetable Matrices

**Fig. 2:**
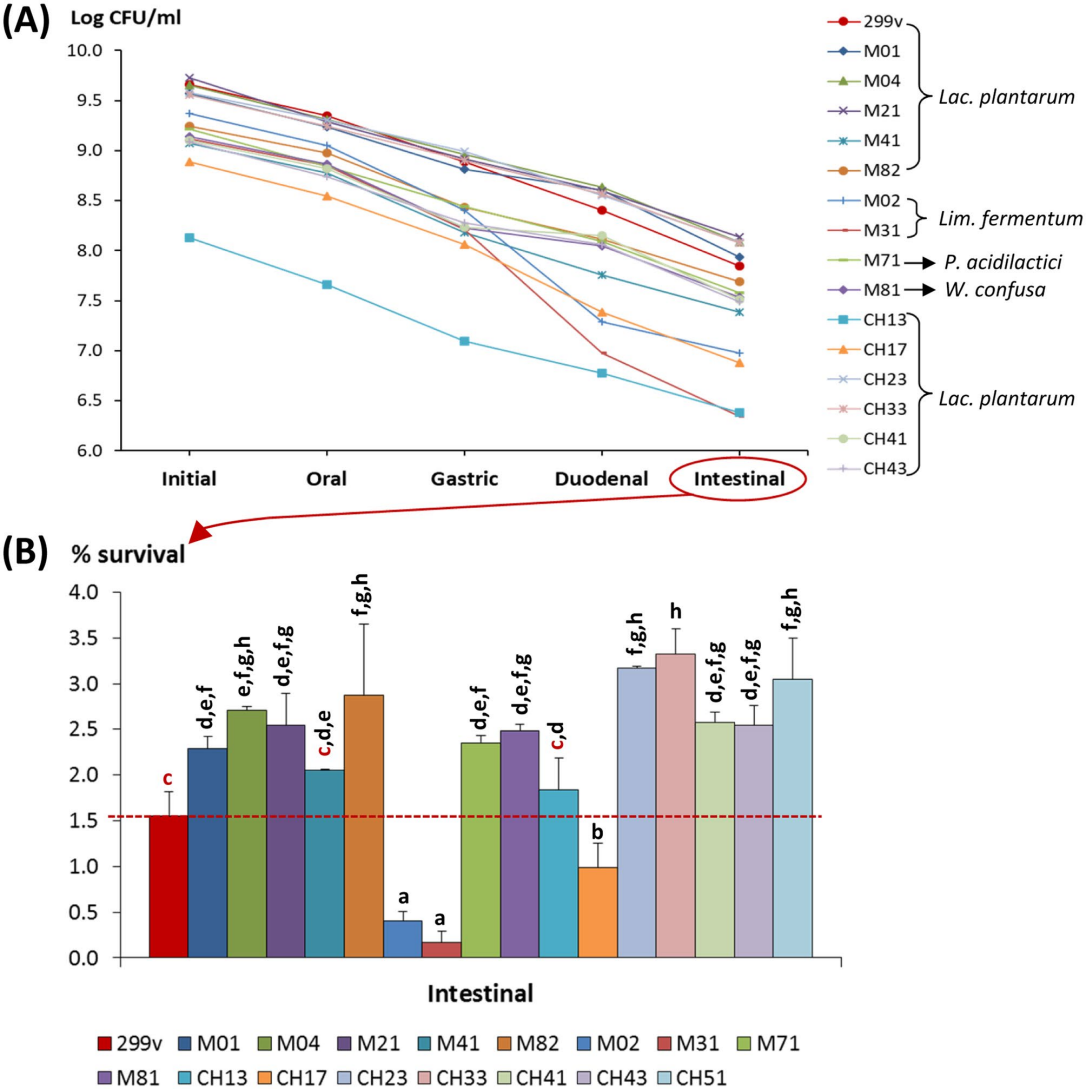
Survival to the static simulated gastrointestinal digestion of the 16 strains isolated in this study and the probiotic *Lac. plantarum* 299v. (**A**) Evolution of the bacterial counts during the sequential steps of the simulation; the coefficient of variation (mean/SD) of the data varied between 1.04 and 24.87%. (**B**) Final survival percentage of the strains (after the intestinal challenge; bars that do not share a common letter are statistically different (*p* < 0.05) according to the SNK mean comparison test

Among the wide variety of biotechnological applications of LAB, one that is receiving huge attention is their use as probiotics for which a high survival to gastrointestinal challenges and/or adhesion capabilities to the colon epithelium are desired traits. In order to select the most robust candidates, the 16 selected strains were subjected to an in vitro simulation of the gastrointestinal transit (GIT) following a standardized static food digestion model [26] adapted to check the survival of these LAB. For comparison purposes, the commercial probiotic *Lac. plantarum* 299v (DSM9843 strain), acquired in the DSMZ-German collection of Microorganisms and Cell Cultures GmbH (Braunschweig, Germany), was submitted to the same procedure. The evolution of the viable bacteria during the sequential GIT steps showed a progressive decline in the number of counts (Fig. 2A). The gastric conditions produced the decrease of 0.5–1.0 log units with respect to the initial point, but the highest reduction was obtained after the introduction of the bile salts (duodenal step). The viability reduction was more drastic for the two *Lim. fermentum* M02 and M31 strains. In fact, after the final GIT challenge, these strains, together with *Lac. plantarum* Ch17, showed a poor percentage of survival being significantly lower than that obtained for the probiotic of reference (Fig. 2B). The remaining strains showed a good performance with a survival comparable, or higher, than the probiotic 299v. Variable results on GIT survival have been obtained by other authors achieving the characterization of novel strains isolated from traditional fermented foods, such as “Shubat” and “Ayran” (dairy products) in Kazakhstan [47], or natural products, sus as “açai” fruits from Brazil [48]. The survival capability is a characteristic associated with strains, but not with species. For instance, the capability of specific *Lac. plantarum* strains to deal with bile salts has been related to the presence of hydrolases able to modify these molecules; this was a feature related to gut-origin strains, but it has been also recently reported for food origin strains [49].

**Fig. 3:**
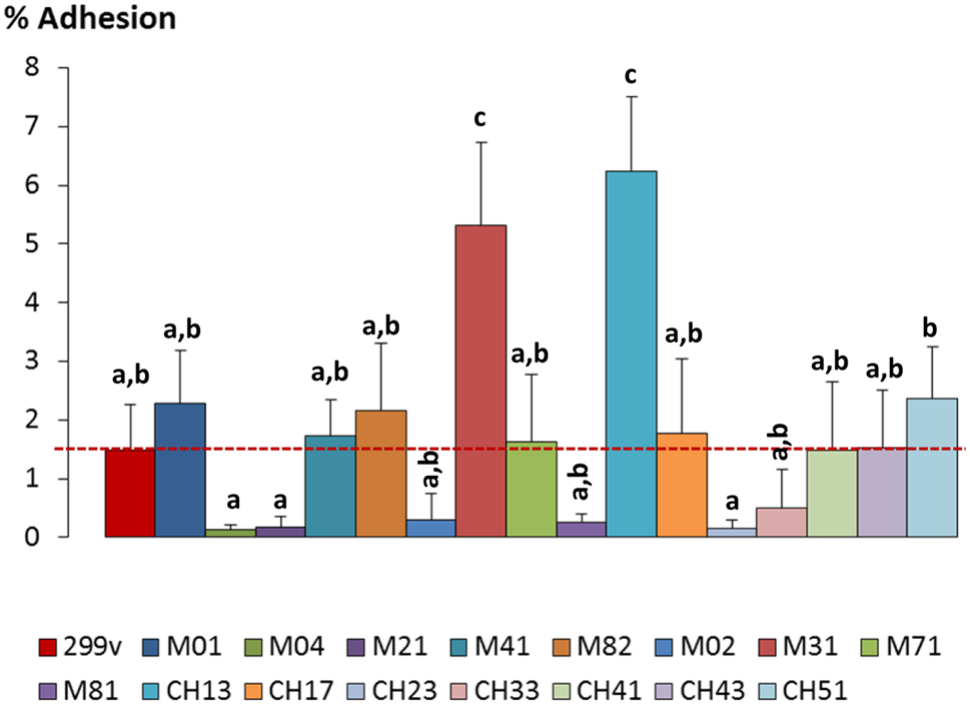
Adhesion percentage (mean and SD) to the human intestinal epithelial cell line HT29 of the 16 strains isolated in this study and the probiotic *Lac. plantarum* 299v. Bars that do not share a common letter are statistically different (*p* < 0.05) according to the SNK mean comparison test

Additionally, the adhesion of the 16 selected strains upon the colonic cell line HT29 was evaluated in order to select those with better, a priori, capability to persist in the colon and therefore to exert a health benefit (Fig. 3). Ten of these strains showed average percentages of adhesion equal or higher than the probiotic strain 299v (1.49 ± 0.76%); remarkably, the strains *Lac. plantarum* Ch13 (6.24 ± 1.27%) and *Lim. fermentum* M31 (5.31 ± 1.42%) presented a notable ability to adhere to colonocytes, which is one of the criteria for the selection of candidates to further characterize their health probiotic benefits, i.e., the probiotic potential. There are many references in the literature reporting the capability of LAB to adhere to the intestinal epithelium [50]; however, the lack of a standardized procedure for quantification of the attached bacteria, as well as for the cellular model to be used, difficult the comparison among different works [51]. This is the reason why is of pivotal relevance to include a probiotic strain of reference, such as 299v, in order to have a criterium for the selection of new potential probiotic candidates. Apart from this bias, it has been reported that the adhesion ability is also a strain characteristic; as an example, among five *Lim. fermentum* strains isolated from fecal samples of Tunisian adult volunteers, only one strain showed three times higher adherence than the probiotic of reference, that in this case was *Lacticaseibacillus rhamnosus* GG, [52]. This capability is related to the presence of specific surface molecules (microorganism-associated molecular patterns or MAMPs) in some strains that are recognized by the specific receptor at the intestinal level [53]. The adhesion ability is an important feature for a probiotic candidate, since it might favor a higher persistence in the intestine, and therefore a prolonged interaction with the host, for instance, at immune level. The adhesion capability is involved in other beneficial effects, such as the antagonism against pathogens through competition for the niche colonization, among others.

In short, in this work, we have highlighted the relevance of homemade fermented foods maintained in the tradition of indigenous communities in a sustainable way, as a source for the isolation of bacteria that could be applied in multiple contexts. From five “Chicha de Siete Semillas” and six “Masato de Yuca” vegetable beverages, a collection of 33 LAB were isolated and identified. Half of them seems to present different genetic fingerprints, as well as specific phenotypic features. The further characterization of the 16 strains was focused on the selection of robust candidates for future probiotic characterization. Following this selection approach, two strains (M31 and Ch13) stand out base on their in vitro capability to adhere to, and probably to persist for a longer period, the large intestine. However, *Lim. fermentum* M31 is not able to face the harsh conditions of the gastric and small intestine transit; then alternative solutions must be studied to improve its performance after the oral administration. On the contrary, *Lac. plantarum* Ch13 is a robust strain, which seems that it could survive in the intestinal environment. This is a candidate to be further studied in order to characterize its potential health benefits.

## Supplementary Information

Below is the link to the electronic supplementary material.

Supplementary file1 (PDF 851 kb)

## Data Availability

Additional data will be made available on reasonable request.
