# Supplementary material for: “Masato de Yuca” and “Chicha de Siete Semillas” Two Traditional Vegetable Fermented Beverages from Peru as Source for the Isolation of Potential Probiotic Bacteria
Source: Probiotics Antimicrob Proteins. 2021 Aug 27;15(2):300–11. doi: 10.1007/s12602-021-09836-x (PMC10024669; doi:10.1007/s12602-021-09836-x)

Supplementary material

Title: "Masato de Yuca" and "Chicha de Siete Semillas" two traditional vegetable fermented beverages from Peru as source for the isolation of potential probiotic bacteria

Authors: Teresa D. Rebaza-Cardenas[1,2], Kenneth Silva-Cajaleón[2], Carlos Sabater[1,3], Susana Delgado[1,3], Nilda D. Montes-Villanueva[2] and Patricia Ruas-Madiedo[1,3,*]

Fig S1. Process flow diagrams followed during the manufacture of the traditional fermented beverages "Chicha de siete semillas" and "Masato de yuca".

Fig S2. RAPD (randomly amplified polymorphic DNA) and REP (repetitive extragenic palindromic sequences) PCR amplification profiles using M13 and BOX-A2R primers, respectively. (A) Profile obtained for LAB isolated from "Chicha de siete semillas" and (B) from "Masato de yuca".

Table S1. Carbohydrate fermentation pattern by means of ApiCH50 (Biomerieux) of the 33 isolates used in this study. A yellow colour means positive (+) fermentation, whereas a slight change of color (indicated by +/-) was not a clear result; the absence of symbol means no fermentation.

Table S2. Enzymatic activities of the 16 strains found in this study measured by Api-zym (Biomerieux). Values greater than or equal to 3, mean a positive reaction.

Fig S3. Hierarchical All-against-All association testing (HAllA) describing the associations between enzymatic activity (vertical axis) and carbohydrate fermentation

profiles (horizontal axis). For this purpose, pairwise Pearson correlation coefficients were calculated ($p_{adj} < 0.05$). Red and blue cells indicate positive and negative correlations, respectively. Colour intensity is in proportion to magnitude.

Fig S4. Principal components analysis (PCA) of enzymatic activities **(A)** and carbohydrate fermentation **(B)** data showing sample distribution according to the producer. **PC**: Principal component.

Fig S1. Process flow diagrams followed during the manufacture of the traditional fermented beverages “Chicha de siete semillas” and “Masato de yuca”.

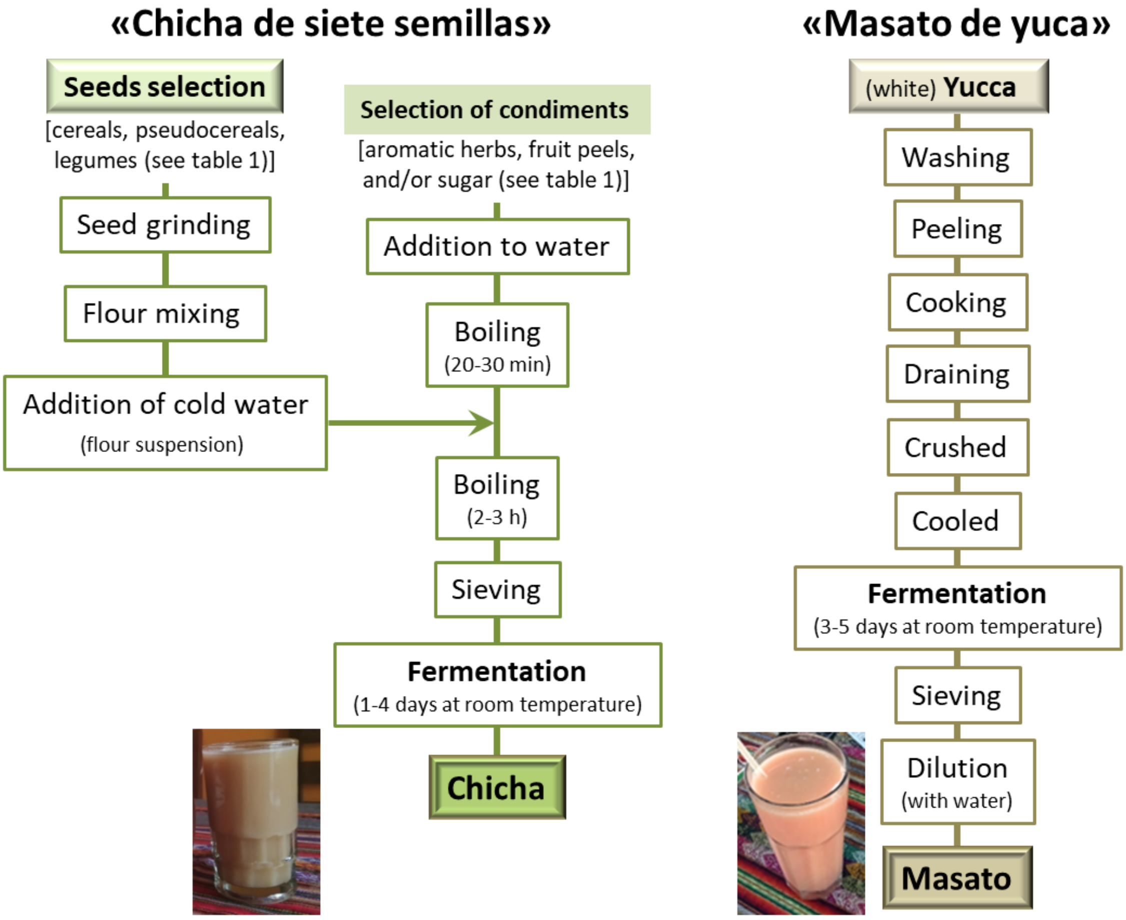

Fig S2. RAPD (randomly amplified polymorphic DNA) and REP (repetitive extragenic palindromic sequences) PCR amplification profiles using M13 and BOX-A2R primers, respectively. (A) Profile obtained for LAB isolated from "Chicha de siete semillas" and (B) from "Masato de yuca".

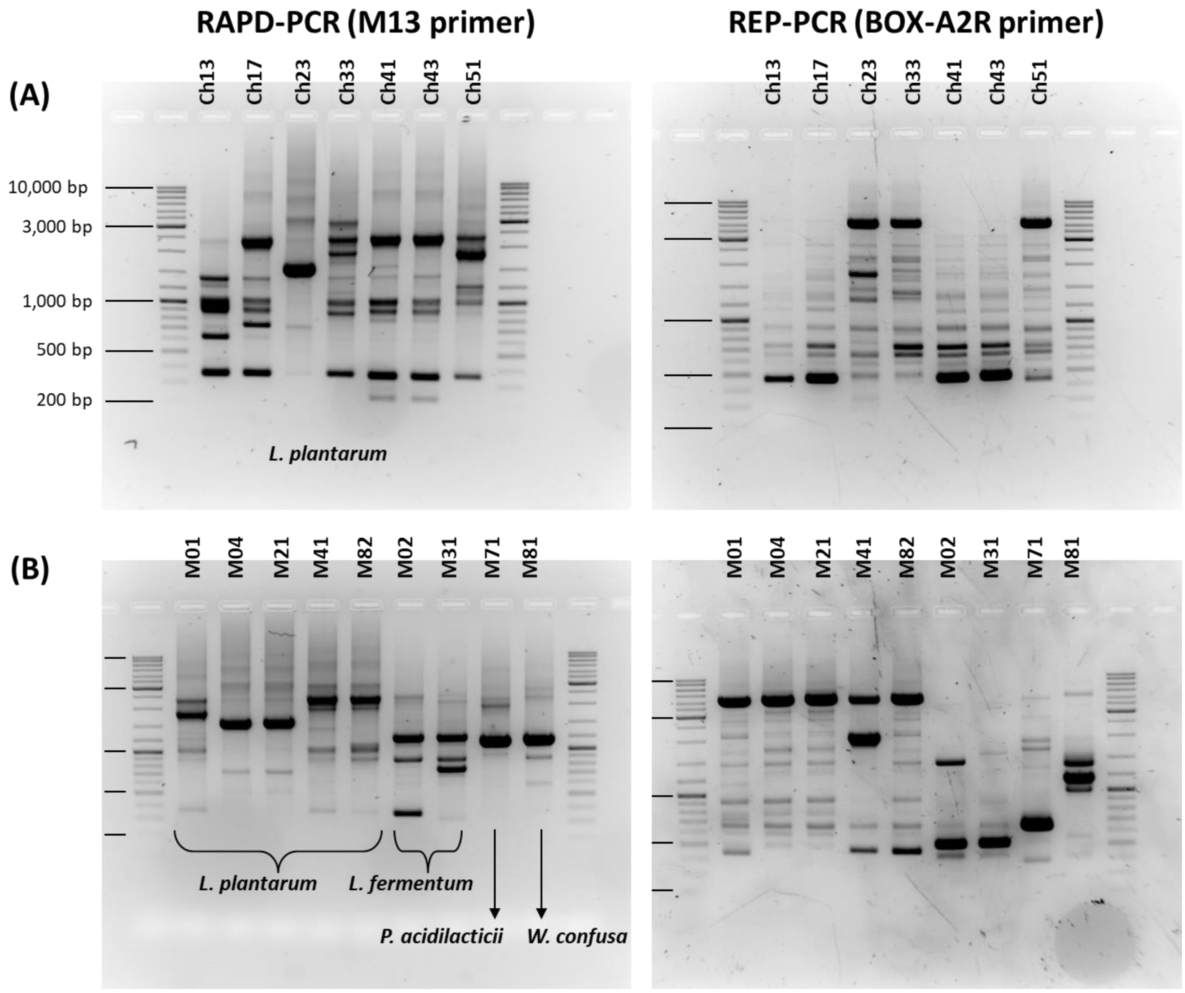

Table S1. Carbohydrate fermentation pattern by means of ApiCH50 (Biomerieux) of the 33 isolates used in this study. A yellow colour means positive (+) fermentation, whereas a slight change of color (indicated by +/-) was not a clear result; the absence of symbol means no fermentation

"Chicha de siete semillas" (*Lac. plantarum*)

Isolate	Glycerol	L-arabinose	D-ribose	D-galactose	D-glucose	D-fructose	D-mannose	D-mannitol	D-sorbitol	Methyl-α-D-mannopyranoside	N-acetylglucosamine	Amygdalin	Arbutin	Esculin	Salicin	D-cellobiose	D-maltose	D-lactose	D-melibiose	D-saccharose	D-trehalose	D-melezitose	D-raffinose	Starch	Gentiobiose	D-turanose	D-arabitol	Potassium gluconate
Ch11		+	+	+	+	+	+	+		+	+	+	+	+	+	+	+	+	+	+	+	+	+		+/-	+	+/-	+/-
Ch12	+/-	+	+	+	+	+	+	+		+	+	+	+	+	+	+	+	+	+	+	+	+	+		+/-	+	+/-	+/-
Ch13		+/-	+	+	+	+	+	+		+	+	+	+	+	+	+	+	+	+	+	+	+	+	+/-	+/-	+		+/-
Ch14	+/-	+	+	+	+	+	+	+		+	+	+	+	+	+	+	+	+	+	+	+	+	+		+/-	+	+/-	+/-
Ch15		+	+/-	+	+	+	+	+	+	+	+	+	+	+	+	+	+	+	+	+	+	+	+		+	+	+/-	+/-
Ch16		+	+	+	+	+	+	+	+	+	+	+	+	+	+	+	+	+	+	+	+	+	+		+/-	+	+/-	+/-
Ch17			+		+	+	+	+	+	+	+	+	+	+	+	+	+	+	+	+	+	+	+		+/-	+		+/-
Ch18	+/-	+/-	+/-	+	+	+	+	+	+	+	+	+	+	+	+	+	+	+	+	+	+	+	+		+/-		+/-	+/-
Ch21	+/-	+	+	+	+	+	+	+	+	+	+	+	+	+	+	+	+	+	+	+	+	+	+	+/-	+/-	+	+/-	+/-
Ch22	+/-	+	+	+	+	+	+	+	+	+	+	+	+	+	+	+	+	+	+	+	+	+	+		+/-	+	+/-	+/-
Ch23		+	+	+	+	+	+	+	+	+	+	+	+	+	+	+	+	+	+	+	+	+	+		+/-	+		+/-
Ch31			+		+	+	+	+	+	+	+	+	+	+	+	+	+	+	+	+	+	+	+/-		+/-	+		+/-
Ch32	+/-	+/-	+	+	+	+	+	+	+		+	+	+	+	+	+	+	+	+	+	+	+	+		+/-	+	+/-	+/-
Ch33		+	+	+	+	+	+	+	+	+	+	+	+	+	+	+	+	+	+	+	+	+	+		+/-	+	+/-	+/-
Ch34		+	+	+	+	+	+	+	+	+	+	+	+	+	+	+	+	+	+	+	+	+	+		+/-	+	+	+/-
Ch41			+	+	+	+	+	+	+		+	+	+	+	+	+	+	+	+	+	+	+	+		+/-	+	+/-	+/-
Ch42	+/-		+	+	+	+	+	+	+		+	+	+/-	+	+	+	+	+	+	+	+	+	+		+/-		+/-	+/-
Ch43	+/-		+	+	+	+	+	+	+		+	+	+/-	+	+	+	+	+	+	+	+	+	+		+		+/-	+/-
Ch51		+	+	+	+	+	+	+	+	+	+	+	+	+	+	+	+	+	+	+	+	+	+		+		+/-	+/-
Ch52	+/-		+	+	+	+	+	+	+		+	+	+	+	+	+	+	+	+	+	+	+	+		+/-		+/-	+/-

Table S1-continuation

"Masato de yuca"

	**Isolate**	Glycerol	L-arabinose	D-ribose	D-galactose	D-glucose	D-fructose	D-mamnose	D-mannitol	D-sorbitol	Methyl-α-D-mannopyranoside	N-acetylglucosamine	Amygdalin	Arbutin	Esculin	Salicin	D-cellobiose	D-maltose	D-lactose	D-melibiose	D-saccharose	D-trehalose	D-melezitose	D-raffinose	Starch	Gentiobiose	D-arabitol	Potassium gluconate
Lac. plantarum	M01	+/-	+/-	+/-	+	+	+	+	+	+	+	+	+	+	+	+	+	+	+	+	+	+	+	+		+/-	+/-	+/-
	M04	+/-	+	+	+	+	+	+	+	+	+	+	+	+	+	+	+	+	+	+	+	+	+	+	+/-	+	+/-	+/-
	M21		+	+	+	+	+	+	+	+	+	+	+	+	+	+	+	+	+	+	+	+	+	+		+		
	M22	+/-	+	+	+	+	+	+	+	+	+	+	+	+	+	+	+	+	+	+	+	+	+	+		+		+/-
	M41		+/-	+	+	+	+	+	+	+		+	+	+	+	+	+	+	+	+	+	+		+		+		
	M82			+	+	+	+	+	+			+	+		+	+	+	+		+	+	+	+			+/-		
Lim. fermentum	M02			+/-	+/-	+	+/-	+	+			+	+/-	+	+	+	+/-	+	+	+	+	+	+	+		+/-		+/-
	M31		+/-			+	+/-	+/-				+/-			+		+	+	+		+	+	+			+/-		+/-
P. acidilactici	M03		+	+	+	+	+	+	+			+		+	+	+	+	+	+	+	+	+	+	+		+/-		+/-
	M32		+	+	+	+	+	+	+/-	+		+	+	+	+	+	+	+	+	+	+	+	+			+/-		
	M33		+	+	+	+	+	+				+	+	+	+	+	+	+	+	+	+	+	+			+/-		
	M71		+	+	+	+	+	+				+	+		+	+	+	+	+	+	+	+	+			+/-		
W. confusa	M81*		+	+	+	+	+	+	+	+		+/-	+	+	+	+	+	+	+	+	+	+	+			+/-		+/-

*D-xylose (+); Potassium 2-ketogluconate (+/-)

Negative (no fermentation) in all isolates for: Erythritol, D-xylose, L-xylose, D-Adonitol, Methyl-β-D-xylopyranoside, L-sorbose, L-rhamnose, Dulcitol, Inositol, Methyl-α-D-glucopyranoside, Inulin, Glycogen, Xylitol, D-lyxose, D-tagatose, D-fucose, L-fucose, L-arabitol, Potassium-2-ketogluconate, Potassium 5-ketogluconate

Lac. plantarum (Lactoplantibacillus plantarum), Lim. Fermentum (Liosilactobacillus fermentrum), P. acidilactici (Pediococcus acidilactici), W. confusa (Weisella confusa).

Table S2. Enzymatic activities of the 16 strains found in this study measured by Api-zym (Biomerieux). Values up to 3, mean a positive reaction.

**Food**	**Species**	**Strain**	Alkaline phosphatase	Esterase (C 4)	Esterase lipase (C 8)	Leucine arylamidase	Valine arylamidase	Cystine arylamidase	Acid phosphatase	Naphthol-AS-BI-phosphohydrolase	α-galactosidase	β-galactosidase	α-glucosidase	β-glucosidase	N-acetyl-β-glucosaminidase
**“Chicha de siete semillas”**	*L. plantarum*	Ch13		1		5	1	1	4	5			5	2	
		Ch17			1	5	1	1	4	5			5	1	
		Ch23	1		1	5	4	2	4	5		4	4	5	4
		Ch33		1		5	4		3	3		5	5	5	5
		Ch41		1	1	4	1		3	3		3	4		
		Ch43				4	3	2	4	4		4	4		
		Ch51	1	1	1	5	4		4	3		2		4	3
**“Masato de yuca”**	*L. plantarum*	M01			1	4	4		5	4		3		4	3
		M04			2	5	4		4	4		4	4	5	4
		M21	1	1	1	5	4		4	4		3	4	5	5
		M41		2	2	5	4		4	4		3		3	2
		M82				4	3		4	4					
	L. fermentum	M02			3	4	1		5	4	5	5	5		
		M31		3	3	4			5	4	2	4			
	P. acidilactici	M71				5	5		3	4					
	W. confusa	M81	2			3	1		5	4					

Negative (no activity) in all strains for: Lipase (C 14), Trypsin, α-chymotrypsin, β-glucuronidase, α-mannosidase, α-fucosidase

Fig S3. Hierarchical All-against-All association testing (HAllA) describing the associations between enzymatic activity (vertical axis) and carbohydrate fermentation profiles (horizontal axis). For this purpose, pairwise Pearson correlation coefficients were calculated ($p_{adj} < 0.05$). Red and blue cells indicate positive and negative correlations, respectively. Color intensity is in proportion to magnitude.

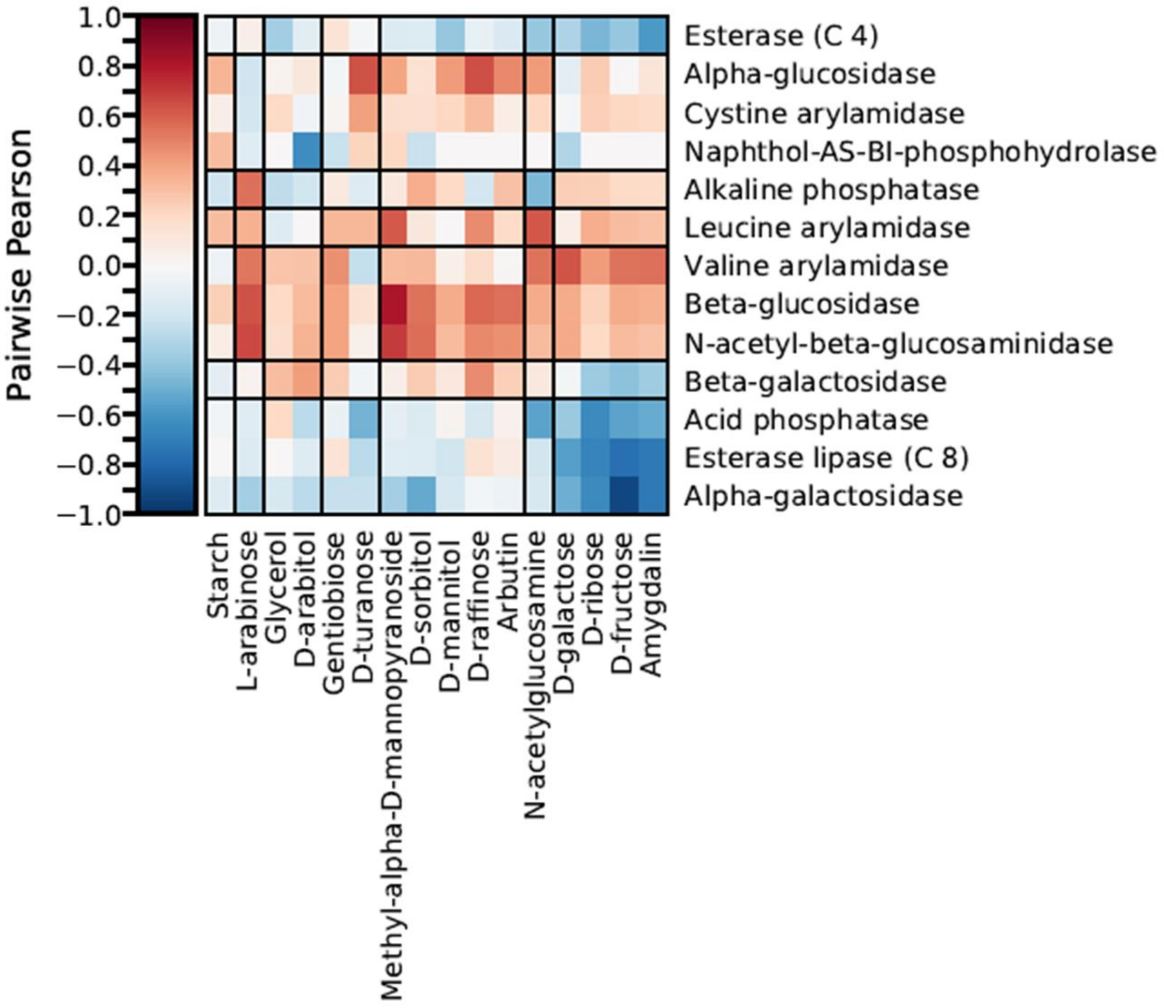

Fig S4. Principal components analysis (PCA) of enzymatic activities **(A)** and carbohydrate fermentation **(B)** data showing sample distribution according to the producer. **PC**: Principal component.

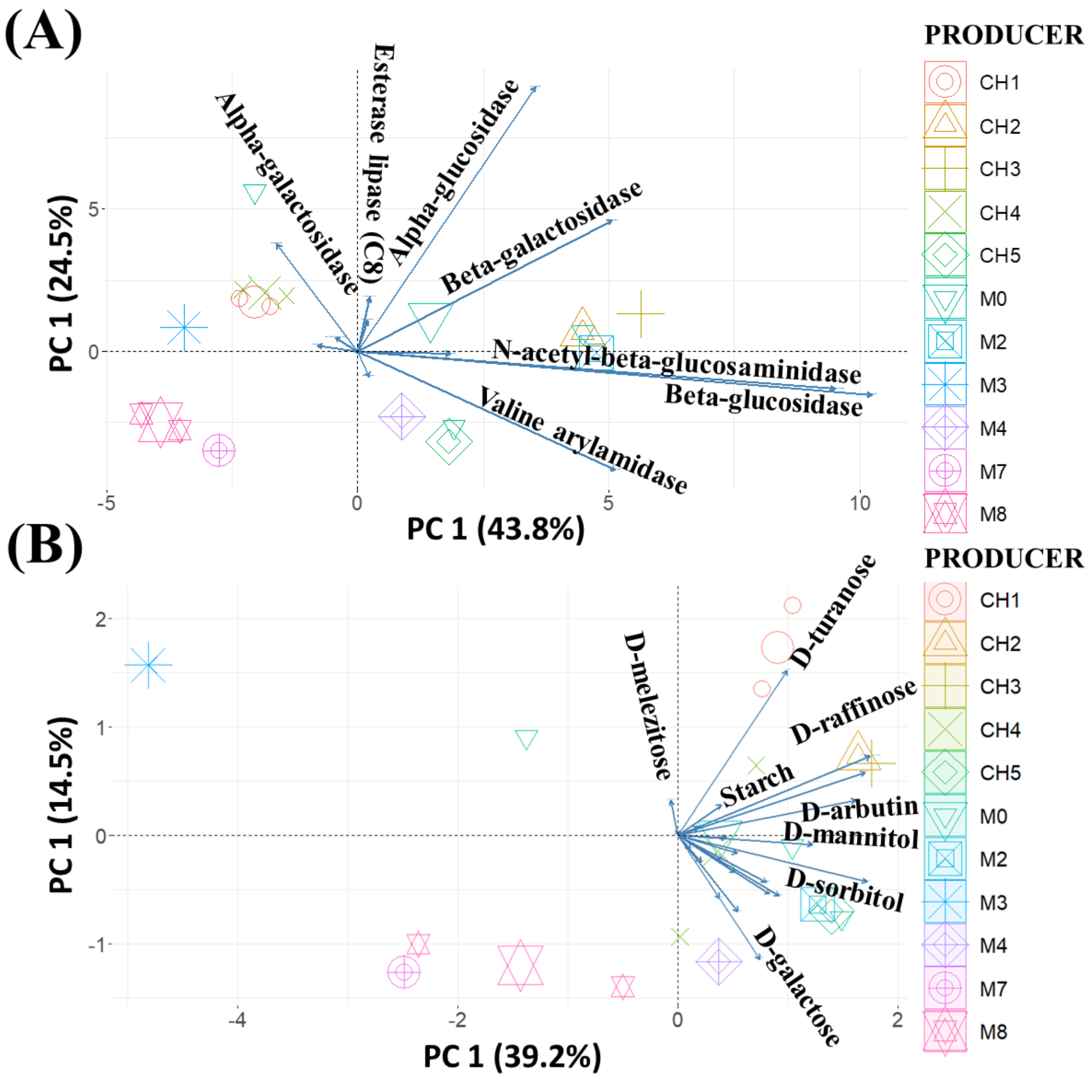

Supplement: Supplementary file 1 — Supplementary file1 (PDF 851 kb) [file 12602_2021_9836_MOESM1_ESM.pdf]
